# Rapid and Replaceable Luminescent Coating for Silicon-Based Microreactors Enabling Energy-Efficient Solar Photochemistry

**DOI:** 10.1021/acssuschemeng.2c03390

**Published:** 2022-08-04

**Authors:** Tom M. Masson, Stefan D. A. Zondag, Michael G. Debije, Timothy Noël

**Affiliations:** † Flow Chemistry Group, van’t Hoff Institute for Molecular Sciences (HIMS), Universiteit van Amsterdam (UvA), Science Park 904, 1098 XH Amsterdam, The Netherlands; ‡ Department of Chemical Engineering and Chemistry, Stimuli-Responsive Functional Materials & Devices, Eindhoven University of Technology, Groene Loper 3, Bldg 14-Helix, 5600 MB Eindhoven, The Netherlands

**Keywords:** photochemistry, energy conversion, luminescent solar concentrator, solar energy, microreactor technologies, luminescent coatings

## Abstract

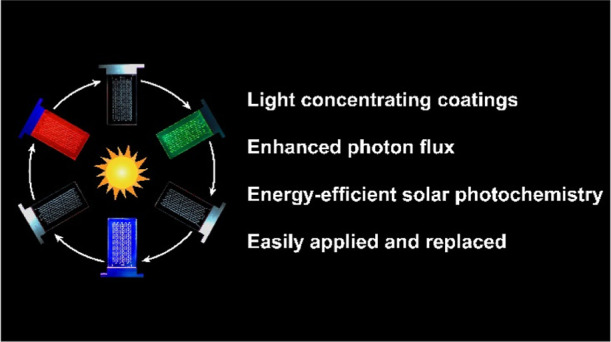

The sun is the most sustainable source of photons on the earth but is rarely used in photochemical transformations due its relatively low and variable intensity, broad wavelength range, and lack of focus. Luminescent solar concentrator-based photomicroreactors (LSC-PMs) can be an answer to all these issues, but widespread adoption is plagued by challenges associated with their complicated manufacturing. Herein, we developed a new strategy to accelerate and ease the production of LSC-PMs by depositing a thin luminescent film on commercially and widely available silicon-based microreactors. The protocol is fast and operationally simple, and the luminescent coating can be easily removed and replaced. This enables rapid tuning of the luminescent coating to fit the requirements of the photocatalytic system and to increase the photon flux inside the microreactor channels.

## Abstract 2

Implementation of a novel method for fast screening of luminescent coatings enables energy-efficient solar photochemistry using conventional silicon-based microreactors.

## Introduction

The sheer complexity of biologically active molecules demands continuous efforts from synthetic organic chemists to develop new and more efficient synthetic strategies.^1^ In addition, spurred by the principles of green chemistry and engineering, there is also a growing desire to develop greener and more sustainable processes.^2−4^ With these boundary conditions in mind, it is only natural that photocatalysis has received significant attention from researchers in both academia and industry, providing benefits that include high selectivity, mild reaction conditions, improved safety, and the use of photons as green and traceless reagents.^5−10^

However, the true transformative potential of photocatalysis resides in the possibility of using the sun as a renewable, free, and perennial source of energy.^11^ Despite this appealing perspective, harvesting sunlight to drive chemical transformations is far from trivial due to low and variable photon intensity (*e.g.*, passing clouds, seasonal variation, and day/night cycles) and broad wavelength distribution, which often gives rise to deleterious photon-induced reaction pathways.^12^ In our ambition to solve these chemical, practical, and technological issues, a luminescent solar concentrator-based photomicroreactor (LSC-PM) was developed.^13^ This reactor concept allows harvesting of the broad spectrum of daylight and conversion of it *via* fluorescence to a narrower-wavelength band, thus matching the fluorescence emission with the energy levels of the photocatalyst. In addition, the photon intensity reaching the reaction channels can be augmented through a wave-guiding effect inside the refractive reactor material.^14^ In combination with self-optimization protocols^15,16^ and solar panels, we were able to develop an autonomous and off-grid solar mini-plant, which enables the production of complex organic molecules using solar light as the sole energy source.^17^

To date, LSC-PM reactors have been mainly fabricated from PDMS^18,19^ or a combination of PMMA and perfluorinated capillaries. Both fabrication processes are labor- and time-intensive, preventing mass production and adoption. Recently, a 3D-printing technique has been developed by Kim and co-workers,^20,21^ but this fabrication method also has its limitations with regard to mass production. As the forerunner in microreactor technology, silicon-based microreactors have been frequently used due to their chemical resistance, transparency, and excellent heat-transfer characteristics.^22,23^ Consequently, silicon-based microreactors have been commercialized and are available in a wide variety of designs and sizes. The material itself is compatible with a number of applications, including flow chemistry,^24^ photochemistry,^25^ microfluidics,^26^ cell biology,^27^ and lab-on-a-chip uses.^28^ In our aim to further increase the utility of the LSC-PM concept, we wondered if we could use commercially available silicon-based microreactors as a foundation to create a silicon-based LSC-PM. As such, standardized flow reactor technology, with known mass and heat transfer characteristics, can be exploited for various solar-driven photochemical applications. Herein, we show that we can indeed coat silicon-based microreactors with a luminescent layer using an operationally simple spin-coating procedure and subsequently turn them into solar-harvesting reactors, which are comparable in efficiency to our previous designs in terms of photon conversion and photon flux. Moreover, as a unique feature of this strategy, the luminescent layer can be easily removed and exchanged, allowing to tune the reactor to the photocatalytic needs and multiple reusing of the base reactor.

## Results and Discussion

### Design of the Silicon-Based LSC-PM

**Figure 1:**
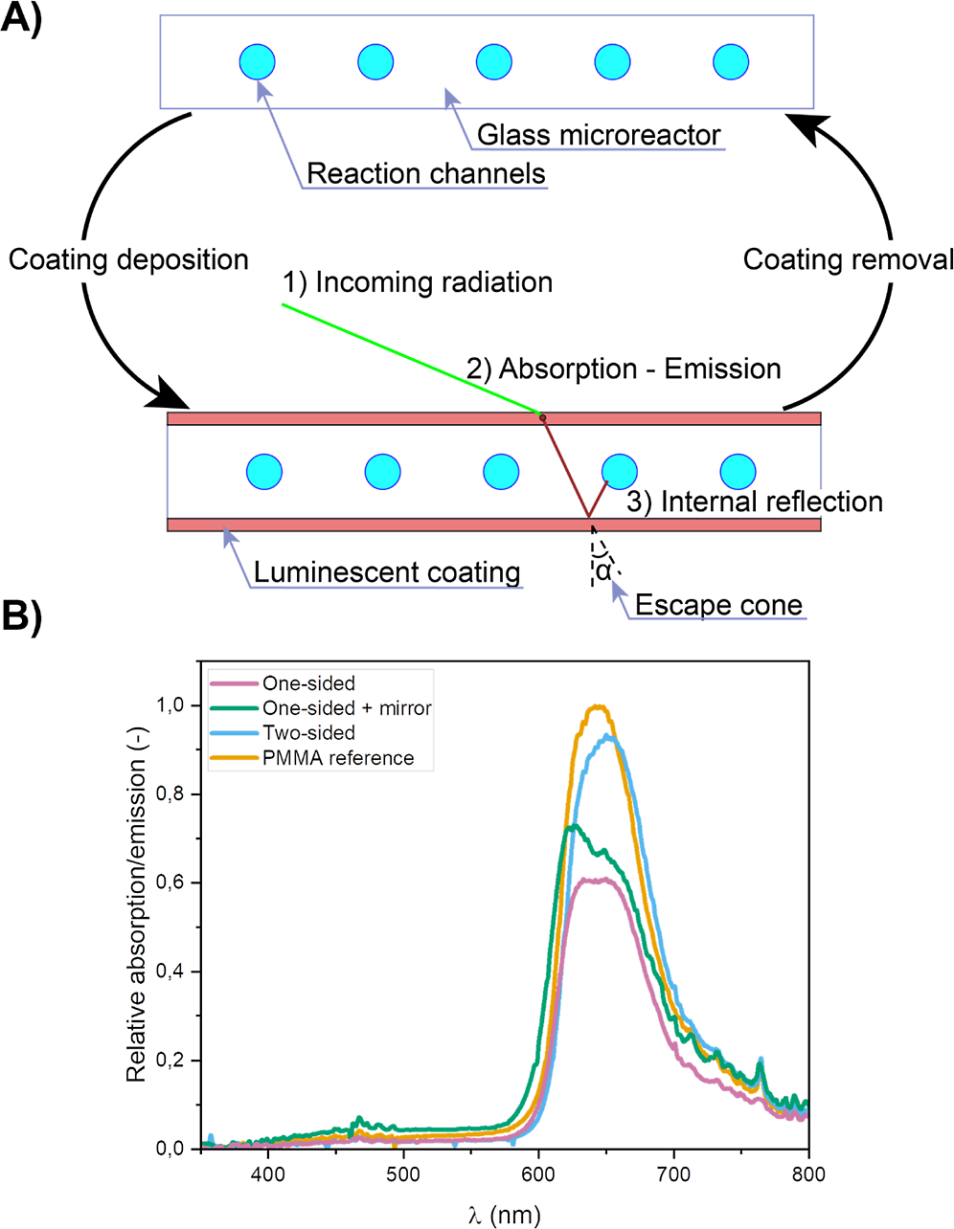
(A) Schematic representation of the application and removal of the luminescent coating to prepare a silicon-based LSC-PM. The deposition of a luminescent coating on the surface of the glass will concentrate photons into the glass microreactor. Different steps: (1) the incident light reaches the coating, (2) the luminescent dye absorbs and re-emits the radiation toward the glass photomicroreactor, and (3) depending on the incident angle of the photon, the light is guided *via* total internal reflection to the reactor channels. (B) Effect of different coating strategies on the edge emission of standard glass substrates; a PMMA reference material is used for comparison with our earlier work.^21^

Our method uses a polymer-based coating which is fixed to the surface of a glass microreactor. By loading a luminescent dye in the polymer, the coating will convert the incoming light and the fluorescent emission will be guided inside the glass reactor, where it can be used to induce photocatalytic transformations (Figure 1A). Such coating methods were previously applied in the energy field to reduce the reabsorption losses of the emitted light by the LSC light guide.^29^ However, in our design, this coating method unlocks flexibility in the design process: instead of designing a microreactor within an LSC matrix, which requires expert knowledge in microchannel design, this approach makes use of commercially available silicon-based microreactors and converts these widely available reactor designs into solar-harvesting ones. Since the coating has a similar refractive index to PMMA (∼1.49), the incident light will be guided to the surface of the glass and enter the silicon-based microreactor. Indeed, with a refractive index of ∼1.47 for Borofloat 33 glass, the light will not be trapped at the glass–coating interface and photon losses can be minimized significantly. In contrast, when the light reaches the coating–air interface, the large difference in refractive index favors internal reflection. Consequently, the photons are waveguided within the reactor material until they reach the reaction channels (see Figure 1A).

The coating material is a result of a photo-induced copolymerization between two acrylate monomers: a base of methyl methacrylate is needed to solubilize both the photoinitiator and the luminescent dye, and dipentaerythritol pentaacrylate is added to strengthen the resulting polymer and to increase the viscosity of the coating. To apply the coating on the glass reactors, two different methods were investigated for their reproducibility and their ease of implementation. Bar coating was first used to layer the coating, but it led to inhomogeneous coatings for both small samples and for larger reactor samples.^30^ Spin-coating was subsequently used to deposit the polymer on the flat substrate surfaces. By modifying the spinning speed and time, a homogeneous coating of uniform thickness could be obtained in a reproducible fashion. The coating was subsequently photopolymerized using UV lamps to form the final coating.

To compare the performances of individual coatings, the edge emissions were determined for standard glass substrates (30 × 30 mm^2^) with the luminescent coatings deposited on the top surfaces. The edge emission measurement quantifies the light escaping from the edge of the substrate while irradiating its top surface with simulated sunlight (AM 1.5G) and can be correlated with the photon flux received inside the reaction channels.^14^ The spectrum captured from the side of the sample is a result of both the downconversion and the waveguiding effect inside the reactor material.^31^

First, the luminescent dye loading inside the deposited coating was investigated. The edge emission measurements demonstrated that a higher dye loading led to greater edge emission because more incident light can be absorbed and released inside the waveguide (see the Supporting Information). This result differs from previous LSC-PM designs, where increased concentrations of luminescent dye resulted in higher reabsorption losses. However, in a coated, silicon-based LSC-PM, reabsorption by the dye can only occur inside the thin coating layer, but not in the 6 mm-thick silicon-based microreactor itself, thus effectively minimizing reabsorption losses. The maximum dye loading of the coating is governed by the solubility in the monomer solution. Increasing the dye loading above this solubility limit will not result in a higher luminescence and will even result in inhomogeneities, causing undesired light scattering and absorption losses.^32^ After testing the solubility and homogeneity of the different dyes and thicknesses, optimal conditions were determined (see the Supporting Information). A thickness of 18 μm resulted in the greatest edge emission, while retaining a homogeneously spread coating layer, and maximum loadings of 1.0, 0.5, and 0.25 wt % were determined for the red dye Lumogen F Red 305 (LR305), the green dye DFSB-K160, and the violet dye Lumogen F Violet 570 (LV570), respectively.

After this coating optimization, the LR305-based coating was compared to a previously designed PMMA sample doped with LR305. As depicted in Figure 1B, different reactor–coating configurations were investigated to maximize the light harvesting capacity. In the first configuration, the luminescent coating was only applied on the top surface (denoted as one-sided coating). This configuration proved to be less effective in harvesting solar energy compared to the PMMA reference. Hence, in the second design, an additional silver coating was applied to the bottom surface of this device to reflect nonabsorbed, transmitted photons back into the device (see the Supporting Information). Unfortunately, the overall measured edge emission was not significantly enhanced by this addition. This observation can be rationalized by the fact that most unabsorbed photons are situated in a wavelength range where neither the device nor the reaction mixture absorbs.^14^ Finally, the optimal result was obtained when the luminescent coating was applied on both sides of the silicon-based microreactor (denoted as two-sided coating). Having two luminescent layers increases the odds of absorption and re-emission inside the light guide, and edge emissions are comparable in profile and intensity relative to the PMMA reference.

**Figure 2:**
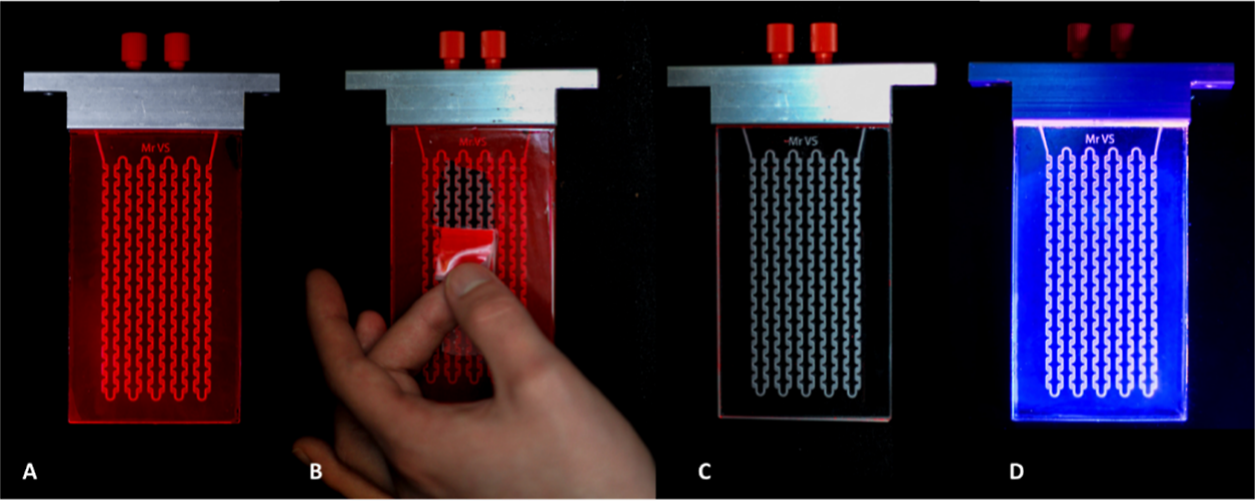
Procedure to exchange the luminescent coating from the silicon-based microreactor: (A) luminescent coating loaded with LR305 on the surface of a glass microreactor. (B) Coating is peeled off with standard clear tape. (C) Microreactor is restored to its original noncoated state. (D) New coating with LV570 dye is applied on the glass surface and shown under UV irradiation.

It is known that different photocatalysts absorb optimally in different wavelength ranges, which is mostly situated around their absorption maxima. This implies that for every photocatalyst, a different reactor must be fabricated to match the emission of the reactor with the absorption characteristics of the photocatalyst. In doing so, the energy efficiency of the solar-driven photocatalytic reaction can be increased significantly. However, it is important to point out that using the method described herein, a suitable luminescent dye can easily be deposited without modifying the standard reactor design (Figure 2A–D). Furthermore, by simply peeling off the coating using adhesive tape (Figure 2B), the original reactor (Figure 2C) can be recovered and immediately reused to layer a new luminescent coating onto the surface (Figure 2D). By doing so, different luminescent dyes (including dye alignment, see the Supporting Information) can be screened in a time- and resource-efficient fashion, allowing selection of the best coating for any photochemical process.

### Reactor Performance

To demonstrate the efficiency of the luminescent coatings for light harvesting, we examined the performance of the silicon-based LSC-PMs in a series of benchmark photocatalytic reactions. From these data, the overall apparent reaction rate constants were extracted and compared.

**Figure 3:**
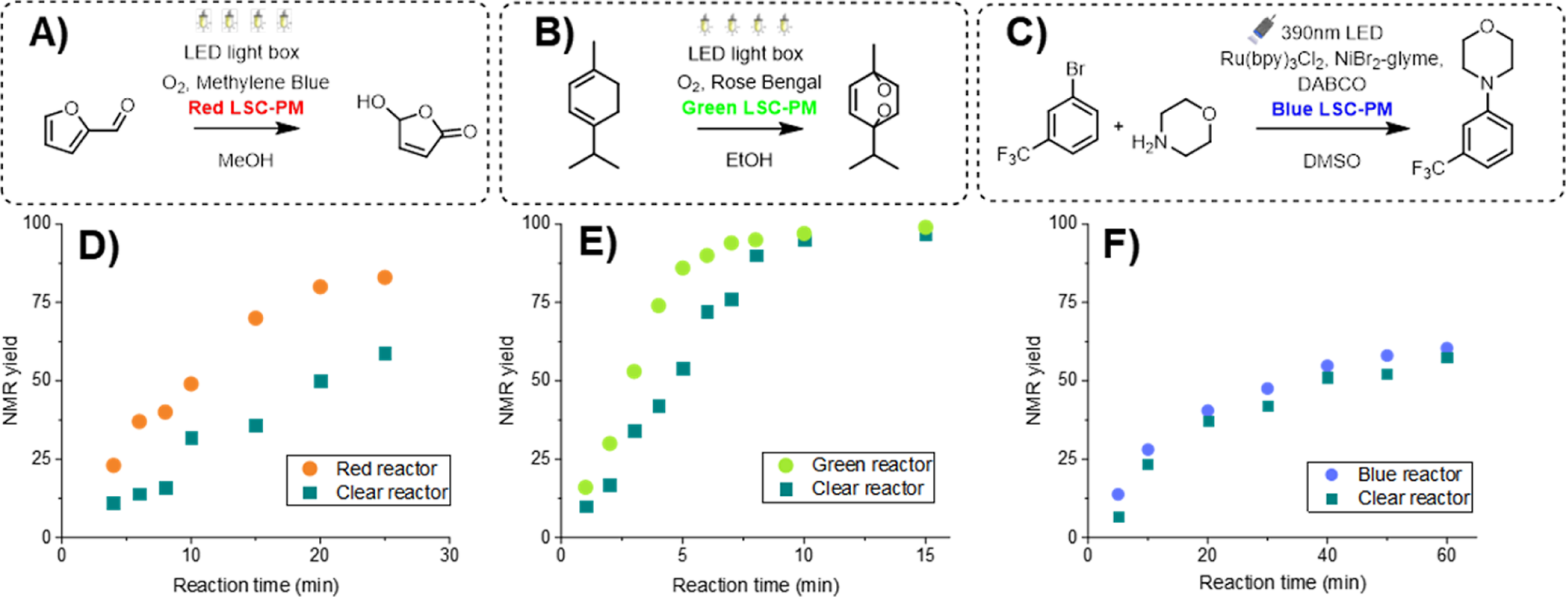
Overview of the reactions carried out in the silicon-based LSC-PMs: (A) furfural oxidation using methylene blue as a photosensitizer. (B) α-Terpinene oxidation using Rose Bengal as a photosensitizer (C) C–N cross-coupling between 1-bromo-3-(trifluoromethyl)benzene and morpholine using Ru(bpy)_3_ as the photocatalyst. (D) Kinetic curves for the furfural oxidation in a red LSC-PM and a noncoated reactor. (E) Kinetic curves for the α-terpinene oxidation in a green LSC-PM and a noncoated reactor. (F) Kinetic curves for the C–N cross-coupling in a blue LSC-PM and a noncoated reactor.

The red LSC-PM, with a coating containing the dye LR305, was evaluated in the oxidation of the biobased platform molecule furfural using methylene blue as the photosensitizer to convert triplet oxygen into singlet oxygen (Figure 3A).^33,34^ Singlet oxygen subsequently reacts with furfural to yield 5-hydroxy-2(5*H*)-furanone. This useful photochemical transformation is the first step in the production of maleic acid,^35^ polymers, and coatings.^36^ Since this transformation is generally photon-limited, increasing the photon flux leads to an improved reaction yield, as depicted in Figure 3D. The reaction rate constant was increased by a factor of ∼2.4 for the luminescent device in comparison to the noncoated reactor.

The green LSC-PM, with a coating containing the luminescent green dye (DFSB-K160), was benchmarked in the [4 + 2] cycloaddition between α-terpinene and singlet oxygen using Rose Bengal as the photosensitizer (Figure 3B). This transformation constitutes the key step in the synthesis of the anthelmintic drug ascaridole.^37^ The kinetic curve of this transformation is shown in Figure 3E, and an enhancement factor of ∼1.7 for the reaction rate constant was observed for the coated microreactor (Figure 3E).

While the green and red region in the visible light spectrum can be used for photocatalysis, blue light has been more widely employed due to the higher energy content present in blue photons and its ability to excite various metal-based photocatalysts, such as the common photocatalyst Ru(bpy)_3_Cl_2_.^38,39^ As a benchmark reaction, we selected the C–N cross-coupling between 1-bromo-3-(trifluoromethyl)benzene and morpholine using ligand-free NiBr_2_ and Ru(bpy)_3_Cl_2_ as the photocatalyst (Figure 3C).^38^ As expected, the coating did not significantly affect the reaction performance, where the reaction rate constant only increased by a factor of ∼1.1 (Figure 3F). This is due to the downconverting nature of the fluorescent dye and, thus, due to the limited fraction of UV light present in the solar spectrum, only small increases in photon flux can be anticipated. Notwithstanding, it should be noted that the blue coating can still be used to avoid selectivity issues as it shields the reaction channels from high-energy UV light. In addition, the edge emission observed in the coated reactor can still be used to couple with a light sensor, which is crucial in our self-optimization protocol required to cope with variable light intensities (*e.g.*, when carried out on a cloudy day).^15^

### Modeling

**Figure 4:**
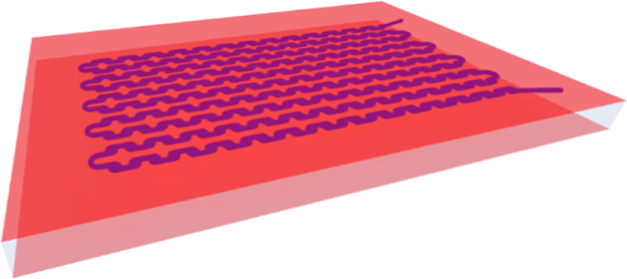
Rendered 3D representation of the two-side-coated reactor.

In order to rationalize, predict, and compare the performance of coated and noncoated commercially available silicon-based microreactors, Monte Carlo ray-tracing simulations were carried out using a modified version of PvTrace.^40^ The implementation of the coating builds upon our previous work on simulating PDMS- and PMMA-based LSC-PMs.^14,17^ The commercial silicon-based reactor was 3D-modeled using the open-source software Onshape and the information provided by the reactor manufacturer (*i.e.*, Little Things Factory). The coating was then represented as a homogeneous thin layer (18 μm) both on the top and bottom surfaces of the reactor (Figure 4).

**Figure 5:**
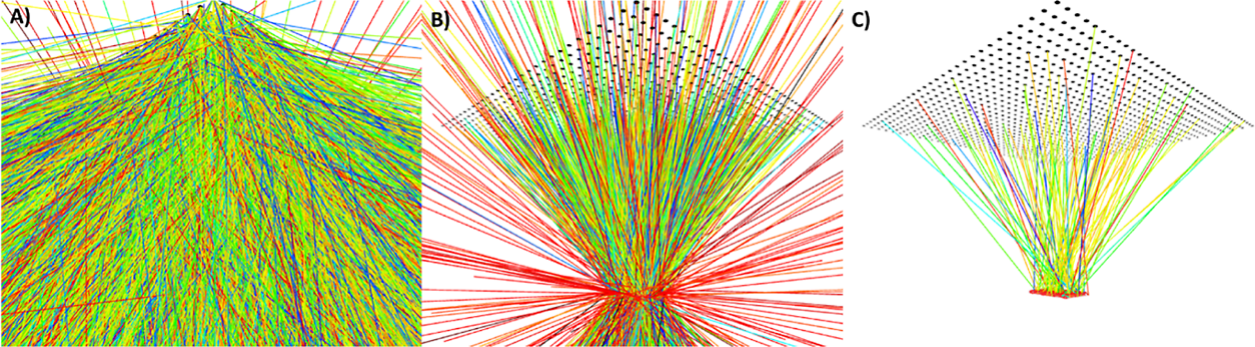
Filtering of LED irradiation in the LightBox simulations. For these images, simulations were run until 100 rays were absorbed by the reaction medium. The ray color represents its wavelength, and the black dots form the LED grid. (A) No filtering: 94 429 rays. (B) Filtering to rays that irradiated the reactor: 1321 rays. (C) Filtered to show solely the rays absorbed by the reaction medium: 100 rays.

To validate the model, a computational representation of the LR305-coated reactor was made to allow comparison with the experimental data. All components in this reactor representation were given the relevant material properties, and the LED-grid (1170 white LEDs, beam angle 66°) of the custom-made LightBox used in the experimental setup was implemented into the code as well. Using this ray-tracing model, the Monte Carlo simulations were performed to calculate the reacted ray fraction of different reactor configurations. The reacted ray fraction is defined here as the number of rays effectively absorbed by the reaction medium divided by the number of rays incident on the reactor surface, effectively filtering out the generated rays that would not reach the reactor. Figure 5 shows this filtering in the MeshCat renderer, yielding a reacted ray fraction of 7.6% in this specific example (value of Figure 5C divided by the value of Figure 5B: 100/1321). The numbers provided in Figure 5 also illustrate that the majority of rays in our setup miss the microreactor (98.6%).

The validation of the model was carried out by running additional simulations until the computed reacted ray fractions converged to a stable value (see the Supporting Information). The reacted ray fractions determined are representative for the efficiency of the reactor devices, and their relative performance can be investigated by comparing these efficiencies. As was found experimentally by looking into the relative reaction rate constants, the LR305-coated reactor performed better by a factor of ∼2.4. The simulations showed a performance enhancement by a factor of ∼2.3 between the noncoated and two-side-coated reactor (3.5 *vs* 7.8%). The experimental and simulation results showed good agreement, where one cause for worse performance in the simulations is expected to be the exclusion of the effects originating from interactions with the LightBox, such as additional reflections from the walls and bottom plate. In reality, even though these are black and primarily absorbing, some reflection is observed, adding light intensity that the model does not account for. To further validate the model, the difference between the noncoated and clear-coated (coating without dye-doping) reactor was investigated and matched the experimental results (3.5 *vs* 3.4%, a relative 3% decrease in the reacted ray fraction compared to the experimentally determined decrease of 2%). This also showed that the enhanced performance was due to the inclusion of the luminescent dye, both experimentally as for the simulations. With this model, the relative performance of the device is also simulated for the two one-side-coated configurations (top-coated and bottom-coated). The performance of these configurations (5.7% top-coated and 6.2% bottom-coated) was lower than the two-side-coated reactor (7.8%), as expected from the measured relative edge emission, shown in Figure 1B, and higher than the noncoated configuration (3.5%). The difference between top and bottom coating can be explained when considering that an incoming ray would potentially have directly reached the channels. For the top-coated configuration, this ray can be intercepted and emitted in a different direction from its initial path, which contributes as a relative loss of irradiance. The bottom coating, however, can intercept otherwise transmitted rays and re-emit them, contributing as a potential relative increase in irradiance. Most importantly, these simulation results showed that the relative performance can accurately be determined using the model, allowing for further screening of parameters such as coating thickness, catalyst and dye concentrations, light sources, the addition of mirrors or white scatterers (see the Supporting Information), geometrical setups, and orientations as long as the properties are representative for the system.

## Conclusions

We have developed a new and operationally simple strategy to convert commercially and widely available silicon-based microreactors into light-harvesting photochemical reactors. The strategy exploits a conventional spin-coating technique to coat silicon-based microreactors with a layer of a luminescent material. Essentially, this method allows one to convert a conventional, transparent microreactor into an efficient luminescent solar concentrator-based photomicroreactor, as demonstrated by both experimental and simulation data. Notably, the coatings can be easily removed and exchanged to match the needs of the different photocatalytic reactions, enabling applications spanning the entire visible light spectrum. While previous LSC-PM designs required some chemical engineering knowledge, such as microreactor design (*e.g.*, to manage mass and heat transfer) and specialized manufacturing techniques, this novel approach is more practical and versatile and separates the reactor design aspects from its solar-harvesting applications. We believe that this fabrication method can be advantageous regarding both the access to bespoke reactors and the mass production of solar-harvesting reactor technology.

## Supporting Information Available

Preparation, deposition, and removal of the coating; edge emission measurements; dye loading and film thickness screening; experimental measurements; determination of the improvement factor; ray tracing simulations; liquid crystal coating; and NMR spectra (PDF)
