# Supplementary material for: Rapid and Replaceable Luminescent Coating for Silicon-Based Microreactors Enabling Energy-Efficient Solar Photochemistry
Source: ACS Sustain Chem Eng. 2022 Aug 4;10(32):10712–7. doi: 10.1021/acssuschemeng.2c03390 (PMC9382670; doi:10.1021/acssuschemeng.2c03390)
Supplement: Supplementary file 1 — sc2c03390_si_001.pdf [file sc2c03390_si_001.pdf]

SUPPORTING INFORMATION

Rapid and Replaceable Luminescent Coating for Silicon-based Microreactors enabling Energy-Efficient Solar Photochemistry

Tom M. Masson[a]ǂ, Stefan D. A. Zondag[a]ǂ, Michael G. Debije[b] and Timothy Noël[a]*

[a] T. M. Massonǂ, ir. S. D. A. Zondagǂ, Prof. Dr. T. Noël, Flow Chemistry Group, van ’t Hoff Institute for Molecular Sciences (HIMS), Universiteit van Amsterdam (UvA), Science Park 904, 1098 XH, Amsterdam, The Netherlands. E-mail: t.noel@uva.nl

[b] Dr. M. G. Debije, Department of Chemical Engineering and Chemistry, Stimuli-responsive Functional Materials & Devices, Eindhoven University of Technology, Groene Loper 3, Bldg 14 – Helix, 5600 MB, Eindhoven, The Netherlands.

ǂ T. M. Masson and S. D. A. Zondag contributed equally to this paper

Number of pages: 18

Number of figures: 15

Number of tables: 1

Contents

Coating preparation 3
Coating deposition method 3
Coating removal method 3
Silver coating deposition method 3
Edge emission measurements 4
Dye loading comparison 4
Thickness screening 4
Experimental section 5
Determination of improvement factor 6
Ray-tracing simulations 7
Aligned dye coating 10
NMR spectra 11
References 18

Coating preparation

To prepare 5 g of the coating, 25 mg of Irgacure 184 (photoinitiator, 0.5 wt%, Ciba) and 1.5 g of methyl metacrylate (Lucite) are mixed with the desired amount of luminescent dye. The suspension is stirred and sonicated until complete dissolution. Then, 3.5 g of dipentaerythritol pentaacrylate (Polysciences) is added and the solution is stirred to reach a homogeneous mixture.

The luminescent dye loadings are limited by the solubility of the dye. The luminescent dyes used in this study were Lumogen F Red 305 and Lumogen F Violet 570 from BASF, and DFSB-K160 from Risk Reactor.

Coating deposition method

A 30 mm × 30 mm × 1.1 mm (or 101 mm x 60 mm x 6 mm microreactor) glass substrate was sonicated in acetone for 15 minutes. The substrate was dried under compressed air and irradiated in an ozone generator for 15 minutes. The clean glass substrate was fixed in the spincoater (see Figure S1, Karl Suss) with vacuum. The liquid monomer coating was deposed at the surface and the sample was spun at 800 rpm for 90 seconds to get a homogeneous layer. The coating was photopolymerized for 10 min under nitrogen flow using low intensity UV radiation from a Philips Home Solaria HB 172 to form a cross-linked, solid film.

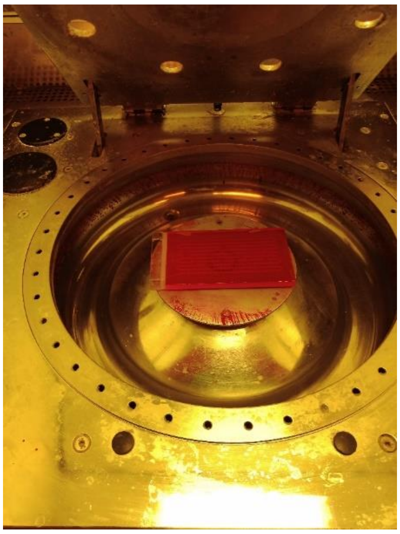

Figure S1. Glass microreactor covered with liquid monomer coating solution in the spincoater.

Coating removal method

Adhesive clear tape was applied at the surface of the coating. By removing the tape, the coating is peeled off the microreactor. After removing most of the coating, the reactor is sonicated in an acetone bath until no trace remains at the glass surface. A new coating can then be applied on the reactor by following the standard coating deposition method.

Silver coating deposition method

To depose a silver layer on the glass substrates, the Tollens reagent is used. First, 1 g of $AgNO_3$ (Sigma-Aldrich) and 1 g of NaOH (TCI) were dissolved in water. When a black precipitate is formed, ammonia is added until complete dissolution. Finally, 4 g of glucose (Sigma-Aldrich) are added and dissolved in the solution. The mixture was poured in a crystallization dish containing the samples to coat. The solution is slowly agitated and heated until the sample is completely coated.

The samples are cleaned with HCl to ensure that only one surface is coated with silver.

Edge emission measurements

The waveguiding efficiency was determined by measuring the edge emission of the previously coated samples. The light escaping from the edge of the samples is proportional to the light received by the reaction channels in the microreactors. Upon irradiation with simulated solar light from a 300 W AM1.5G solar simulator light source (Lot Oriel Group), edge emissions of the lightguides were measured by an SLMS 1050 integrating sphere (Labsphere) with a diode array detector (RPS900, International Light).

Dye loading comparison

To optimize the dye loading in the coating, several coatings were deposed on model glass substrates. The edge emission of those samples was compared to obtain an optimal value. In Figure S2A and Figure S2B, the loading of Lumogen F Red 305 and DFSB-K160 were screened. The optimal concentration here are 1 wt% and 0.5 wt% respectively, because a higher dye loading decreases the edge emission of the sample. This diminution can come from more reabsorption losses in the film, but the main limiting factor is the solubility of the dye. Increasing the loading further leads to inhomogeneities in the film and aggregates of dye in the polymer. In the case of Lumogen F Violet 570 (Figure S2C), changing the dye loading did not significantly improve the edge emission of the glass. Moreover, the emission is lower than the other coatings because the main absorption band of the dye lies below 400 nm. A solar simulator spectra contains a small component of UV radiations leading to a smaller emission in the blue.

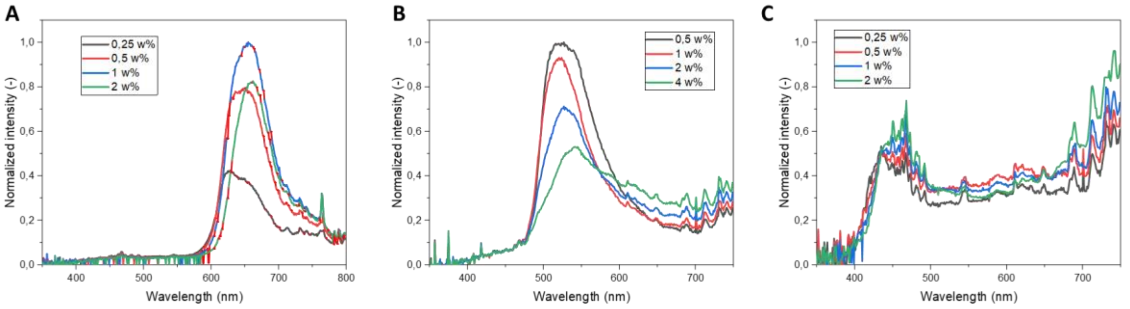

Figure S2. Edge emission of the model glass substrate depending on the luminescent dye loading in the coating. A) Lumogen F Red 305. B) DFSB-K160. C) Lumogen F Violet 570.

Thickness screening

The thickness of the coating was optimized to combine a good homogeneity of the coating and good lightguiding properties. To modify the film thickness, different spinning speeds were investigated while keeping other parameters of the spincoater constant (acceleration = 5 rpm per second, time = 90 seconds). The rotation speed was set at 400 rpm, 800 rpm, 1600 rpm and 3200 rpm, which resulted in thicknesses of, respectively, 27 µm, 18 µm, 11 µm and 9 µm. The thicknesses were determined by averaging 3 measurements of the same sample analyzed by a Fogale Zoomsurf 3D optical profiler. As depicted on Figure S3, the thicker the coating, the greater the edge emission. However, at 27 µm thickness, the coating deposed was no longer homogeneous, as shown on Figure S3B. Hence, the coating applied on the glass microreactors was chosen to be 18 µm.

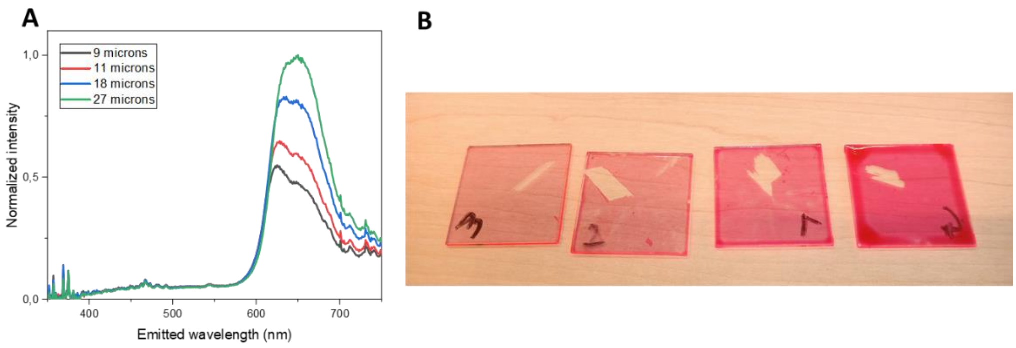

Figure S3. A) Relative edge emission intensity depending on the coating thickness. B) Coated samples of increasing thickness, from left to right: 9 µm, 11 µm, 18 µm, 27 µm. The clear, non-coated, patches on the substrate are used to determine the layer thickness.

Experimental section

Unless explicitly stated otherwise, the experiments were executed in our previously reported custom-made LightBox, consisting of arrays of white LEDs to emulate solar irradiation.[1,2] The experiments were executed using a 1.1 mL glass microreactor purchased from Little Things Factory.

Furfural oxidation

A stock solution was prepared using furan-2-carbaldehyde (240.2 mg, 207 µL, 1 eq., 2.5 mmol, Sigma-Aldrich), p-xylene (265.4 mg, 308 µL, 1 eq., 2.5 mmol, TCI; used as internal standard) and methylene blue (31.99 mg, 0.040 eq., 100.0 µmol, TCI) in MeOH (25 mL, Biosolve). This reaction mixture is mixed with an oxygen flow (1:5 L:G volumetric ratio) to homogeneously fill a 25 mL sample loop (Figure S4A). The biphasic mixture is then pushed with solvent through the glass microreactor (see Figure S4B) inside the LightBox. Fractions with different residence times were collected at steady state, after waiting 3 residence times, and analyzed by quantitative ^{1}H-NMR (300 MHz, $CDCl_3$).

Alpha-terpinene oxidation

A stock solution was prepared using α-terpinene (378 mg, 452 µL, 90 wt%, 1 eq., 2.5 mmol, Sigma-Aldrich) and Rose Bengal (5.1 mg, 0.002 eq., 5.01 µmol, Sigma-Aldrich) in EtOH (25 mL, Biosolve) with trichloroethylene (328 mg, 225 µL, 1 eq., 2.5 mmol, Sigma-Aldrich) as internal standard. This reaction mixture is mixed with an oxygen flow (1:5 L:G volumetric ratio) to homogeneously fill a 25 mL sample loop. The biphasic mixture is then pushed with solvent through the glass microreactor inside the LightBox. Fractions with different residence times were collected at steady state, after waiting 3 residence times, and analyzed by quantitative ^{1}H-NMR (300 MHz, $CDCl_3$).

Morpholine arylation

$Ru(phen)_3Cl_2$ (1.15 mg, 0.0006 eq., 1.8 µmol, Sigma-Aldrich), Triethylenediamine (606 mg, 566 µL, 1.8 eq., 5.4 mmol, Sigma-Aldrich) and 2,3-difluoro benzene (342 mg, 296 µL, 1 eq., 3.0 mmol, Sigma-Aldrich; used as an internal standard) were dissolved in DMF (50 mL, Sigma-Aldrich) in a 25 mL vial. After sparging the solution 3 times with nitrogen, 1-bromo-3-(trifluoromethyl)benzene (675 mg, 1 eq., 3.0 mmol, TCI) and morpholine (392 mg, 389 µL, 1.5 eq., 4.5 mmol, Sigma-Aldrich) were added. The reaction mixture was injected with a syringe pump through a glass microreactor irradiated with a 390

nm Kessil lamp. Fractions with different residence times were collected at steady state, after waiting 3 residence times, and analyzed by quantitative ^{19}F-NMR (400 MHz).

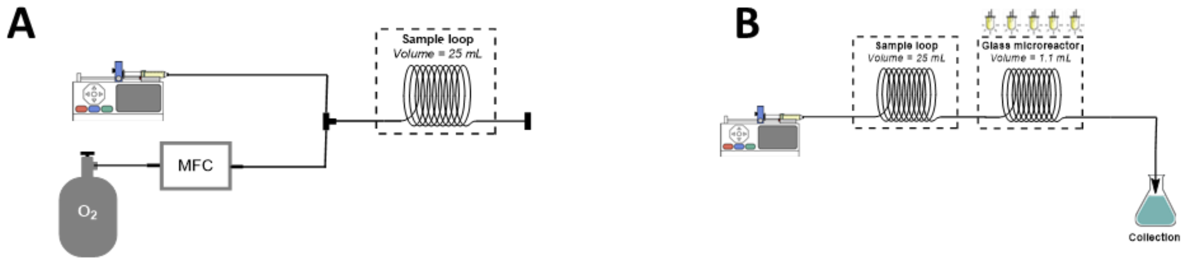

Figure S4. A) Loop filling method to store the reaction mixture. B) Reaction setup for furfural oxidation and alpha-terpinene oxidation

Control experiment

To investigate whether the application of a clear coating has a detrimental effect to the reactor, or whether application of this clear coating alone already provides an enhanced photon flux, control experiments were performed using a non-coated, clear-coated (without loaded dye) and dye-coated reactors. For this, the α-terpinene oxidation under standard conditions was used as a benchmark reaction. To create a transparent coating at the surface of the microreactor, the standard coating procedure was followed without loading any luminescent dye.

Figure S5 shows the kinetic curves for the three device configurations. Comparing the clear coating to the non-coated reactor shows no significant difference in yield. This means that the polymer deposited on the glass does not significantly influence the light-guiding properties of the system. However, the reactor coated with the luminescent dye shows a great enhancement in yield compared to the other configurations. This proves that the luminescent dye is the key parameter with this approach, and not caused by the polymer used for the coating on its own.

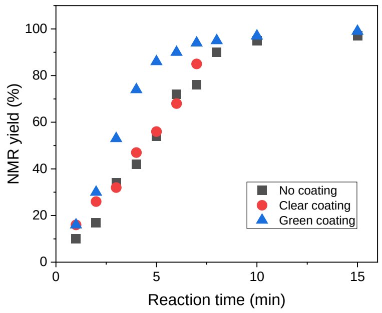

Figure S5. NMR yield of alpha-terpinene oxidation in a non-coated reactor, a clear-coated reactor and a reactor coated with loaded DFSB-K160 luminescent dye.

Determination of improvement factor

For the determination of the improvement factor, or the added benefit of the coatings to the performance of the glass reactors, an unbiased method that takes into account all generated data had to be considered to circumvent unintentionally selecting specific datapoints to exaggerate the overall improvement. To

achieve this, overall molar balances for the reactions were constructed under the assumption of ideal PFR behaviour and simple first order $A \rightarrow B$ reaction kinetics (Eq. S1 and Eq. S2). Even though the reactions are not performed in single-phase, the Taylor flow grants the possibility for the approximation of each liquid slug to be considered as a micro-batch reactor passing through the reactor, allowing the usage of a PFR model due to the equivalence of a micro-batch slug to the infinitesimal volume considered in the integration.

$$\frac{dN_A}{dt} = \Phi_V C_A|_z - \Phi_V C_A|_{z+dz} - kC_A dV \qquad \text{(Eq. S1)}$$

$$\frac{dN_B}{dt} = \Phi_V C_B|_z - \Phi_V C_B|_{z+dz} + k_1 C_A dV \qquad \text{(Eq. S2)}$$

To differentiate between conversion and yield, the assumption was made that both the desired and undesired product formation originates from competitive first order reactions of the substrate; k_1 for the desired product and k_2 for any other undesired reaction (see Eq. S3).

$$k = k_1 + k_2 \qquad \text{(Eq. S3)}$$

From these molar balances, generic equations for the conversion of A and the yield of B can be constructed (Eq. S4 and Eq. S5) and fitted using experimental data to obtain reaction rate constants that can be compared for the non-coated, dye-coated and clear-coated reactors using non-linear curve-fitting.

$$X(\tau) = 1 - \exp\left(-k\tau\right) \qquad \text{(Eq. S4)}$$

$$Y(\tau) = \frac{k_1}{k} \cdot (1 - \exp(-k\tau)) \qquad \text{(Eq. S5)}$$

This method is similar in approach to solely considering the initial reaction rate in the apparent linear region, with the important distinction that the linear curve-fitting of the entire dataset is less prone to overestimation of the apparent reaction rate constant. The resulting reaction rate constants and selectivities towards the desired products in terms of reaction rate constants are given in Table S1.

Table S1. Fitted first order reaction rate constants and selectivities for the experimental data.

	Furfural oxidation		α-terpinene oxidation			Morpholine arylation	
	Non-coated	Red-coated	Non-coated	Clear-coated	Green-coated	Non-coated	Blue-coated
k_1 (1/min)	0.039	0.092	0.196	0.191	0.328	0.0173	0.0198
k_2 (1/min)	0.016	0.020	0.001	0.015	0.002	0.0012	0.0012
k (1/min)	0.054	0.112	0.196	0.206	0.330	0.0185	0.0210
Selectivity (%)	71.2	82.2	99.7	92.9	99.4	93.6	94.4

Ray-tracing simulations

For the Monte Carlo ray-tracing simulations, the glass reactor device was created as a rectangular slab (101 mm x 60 mm x 6 mm). The 3D-modelled reactor geometry was imported as a triangular mesh and placed within the rectangular slab to create the filled reactor channels within the glass reactor device. The coating was then generated as a homogenous thin rectangular slab (101 mm x 60 mm x 18 µm),

duplicated and placed both at the top and bottom surface of the previously constructed reactor representation.

All components of this reactor representation were given the relevant material properties, such as absorption and emission spectra, quantum yield of the luminescent dye, background absorption and refractive indices. Next, the custom-made LightBox was implemented into the code. The grid, consisting of 1170 white LEDs, was generated at the correct experimental height (29.8 cm) and a function was implemented to simulate individual rays from these LED positions according to the experimentally determined emission spectrum and beam angle (66°). The spectra used are shown in Figure S6.

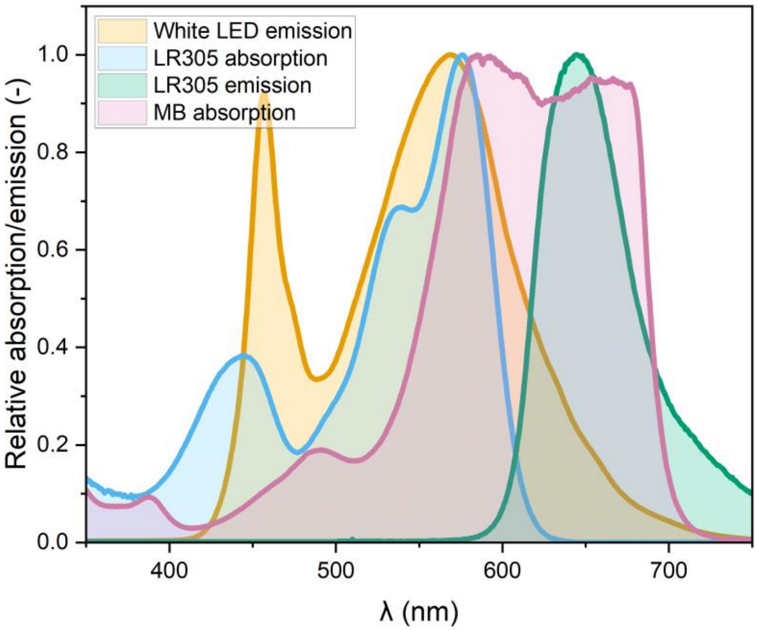

Figure S6. Relative absorption and emission spectra for the relevant components in the simulations.

Using this ray-tracing model, the Monte Carlo simulations were performed until the computed reacted ray fractions (p) converged to a stable value (Figure S7). The simulations were performed until a set target of reacted rays ($N_{reacted}$) was reached, after which this was related to the total amount of rays that interacted with the reactor (N_{total}) to calculate the reacted ray fraction. The convergence to a stable value was monitored by computing the margin of error for proportions (MoE). The reacted ray fraction is considered the sample proportion ($\hat{p}$, Eq. S6) with a sample size of N_{total}. For a confidence interval of 95%, $z = 1.96$ was used to compute the MoE (Eq. S7). The simulations were performed for increasing reacted ray targets until twice the MoE was smaller than 5% of the proportion (Eq. S8), resulting in an approximate confidence of 95% that the actual value of p is within $0.975 \cdot \hat{p} \leq p \leq 1.025 \cdot \hat{p}$.

$$\hat{p} = \frac{N_{reacted}}{N_{total}} \qquad \text{(Eq. S6)}$$

$$MoE = \pm z \cdot \sqrt{\frac{\hat{p}(1-\hat{p})}{N_{total}}} \qquad \text{(Eq. S7)}$$

$$MoE < \frac{0.05 \cdot \hat{p}}{2} \qquad \text{(Eq. S8)}$$

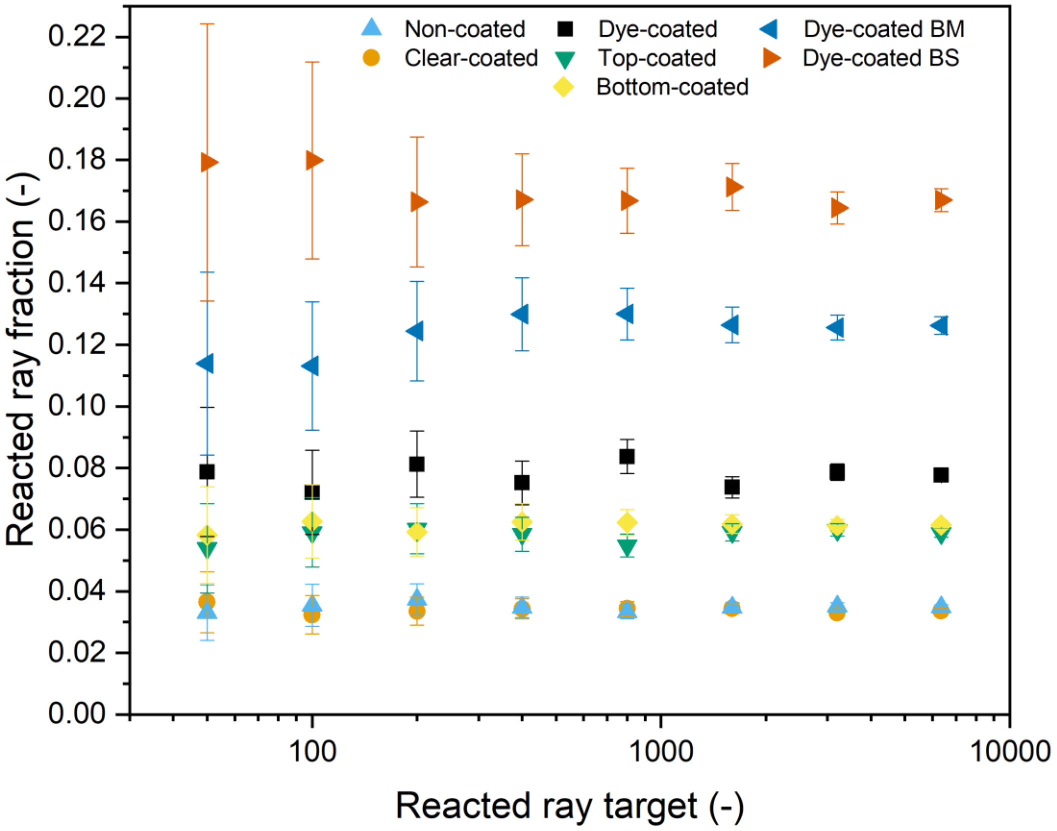

Figure S7. Reacted ray fractions including the decreasing MoE for varying reactor-coating configurations. Unless explicitly stated otherwise, coatings are applied to both the top and bottom side of the reactor. The configurations with BM and BS indicate bottom-mirror and bottom-scatterer, respectively.

Additional simulations were performed for the case where an ideal mirror or perfect white scatterer is placed underneath the reactor. These simulations do not take into account absorption losses by the mirror or scatterer. The mirror is simulated by ensuring that all rays hitting the bottom surface follow ideal specular reflection, fully disabling further Fresnel refraction and transmission. For the white scatterer, all rays that would be transmitted (Fresnel refraction through the bottom surface within the escape cone) normally, are scattered upward with an ideal Lambertian distribution, but otherwise reflected inward with specular reflection just as trapped light normally would in the device.

As expected, the mirror is less effective than the white scatterer, since the light reflected by the mirror that would otherwise transmit through the device is still within the escape cone when it would hit the top surface. This does effectively double the path length within the device, resulting in an extra pass and chance to hit the reaction channels, or be absorbed by the dye. The effectiveness of the mirror also depends on the wavelength of the reflected light and whether it can be absorbed by the dye.

The white scatterer outperforms the mirror, since the scattering randomizes the direction, making it possible for ray directions within the escape cone to be scattered away.

The effectiveness of both the mirror and scatterer is strongly dependent on the light intensity that reaches these surfaces: the “transmitted” light. For thicker coatings, higher dye concentrations, more strongly absorbing materials and a higher reaction channel density their effect would diminish greatly. These results only indicate the potential of their inclusion, but are expected to be less impactful experimentally, where their performance would include losses that have been neglected here.

Aligned dye coating

A 5 wt% PVA in water solution was applied to the top of the glass plates and spun at 1000 rpm for 30 seconds, and the water evaporated at 90 °C for a few minutes. One of the two layers was rubbed on a velvet cloth to form a planar alignment layer.

0.4 wt% of a coumarin derivative dye, 1 wt% photoinitiator (Irgacure 184 (Ciba)), and 1% of the surfactant ((2-(n-ethylperfluorooctanesulfonamido)-ethyl methacrylate stabilized, Acros) were added to LC (Paliocolor LC 242, BASF); all solids were dissolved in Xylene (Biosolve) (45:55 weight ratio solid: solvent) and spin-cast on top of the PVA layers at 1000 RPM for 30 seconds. The devices were then placed on a hotplate at 85 °C for 15 seconds to evaporate the solvent and assist LC alignment and subsequently photocured for 10 minutes under nitrogen flow using low intensity UV radiation from a Philips Home Solaria HB 172 to form a cross-linked, solid film.

The reactor with aligned liquid crystals (order parameter S=0.40) was compared with a reactor coated with no alignment layer (S=0.13). This approach should determine whether intentionally directing the light towards the reaction channels can have a beneficial effect on the reaction conversion as it does on light emission directionality in standard LSC devices.[3,4] The reaction used to compare both coatings was the photo-oxidation of the alpha-terpinene, the standard protocol is described above. However, as shown on Figure S8B, no obvious enhancement can be observed due to the alignment of the liquid crystals. This suggests the potential advantage of light directionality is minimized by short distances between channels: it is anticipated the effects of dye alignment become increasingly visible as the distance between reaction channels increases.

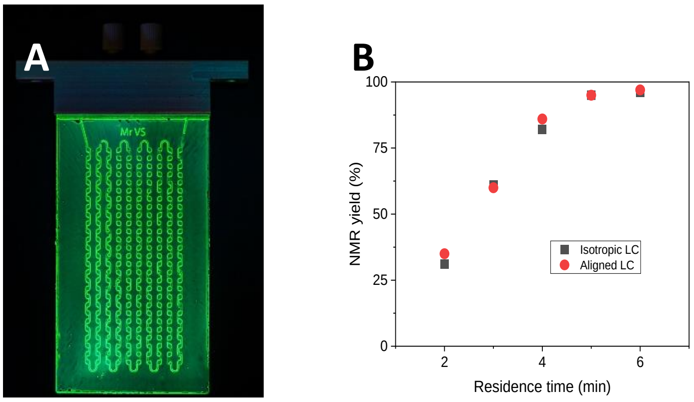

Figure S8. A) Picture of a thin film of liquid crystals embedded with a coumarin derivative dye at the surface of a glass microreactor. B) NMR yield of alpha-terpinene oxidation in glass microreactors coated with isotropic/aligned LC

NMR spectra

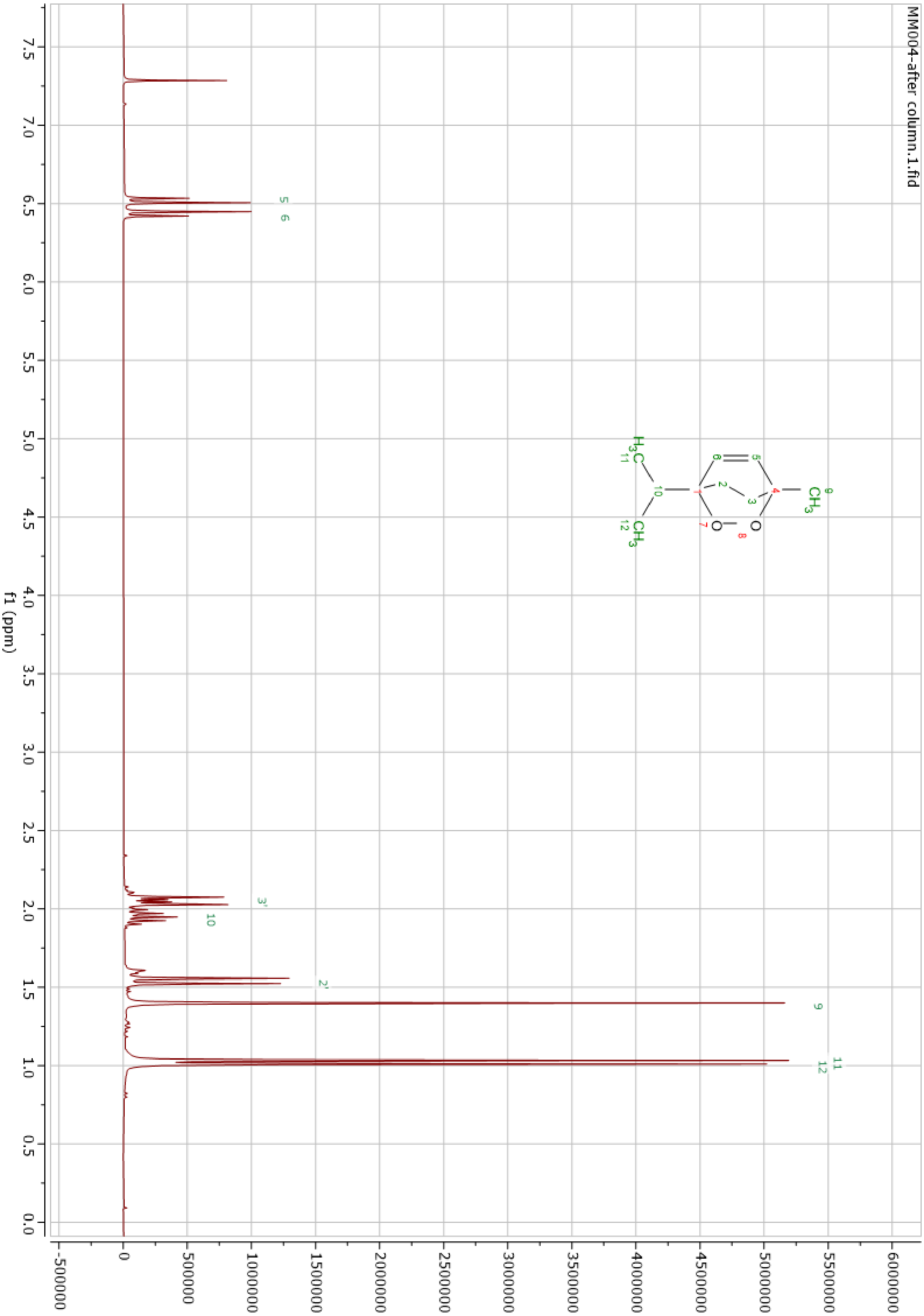

Figure S9: ^{1}H-NMR spectrum of alpha-terpinene

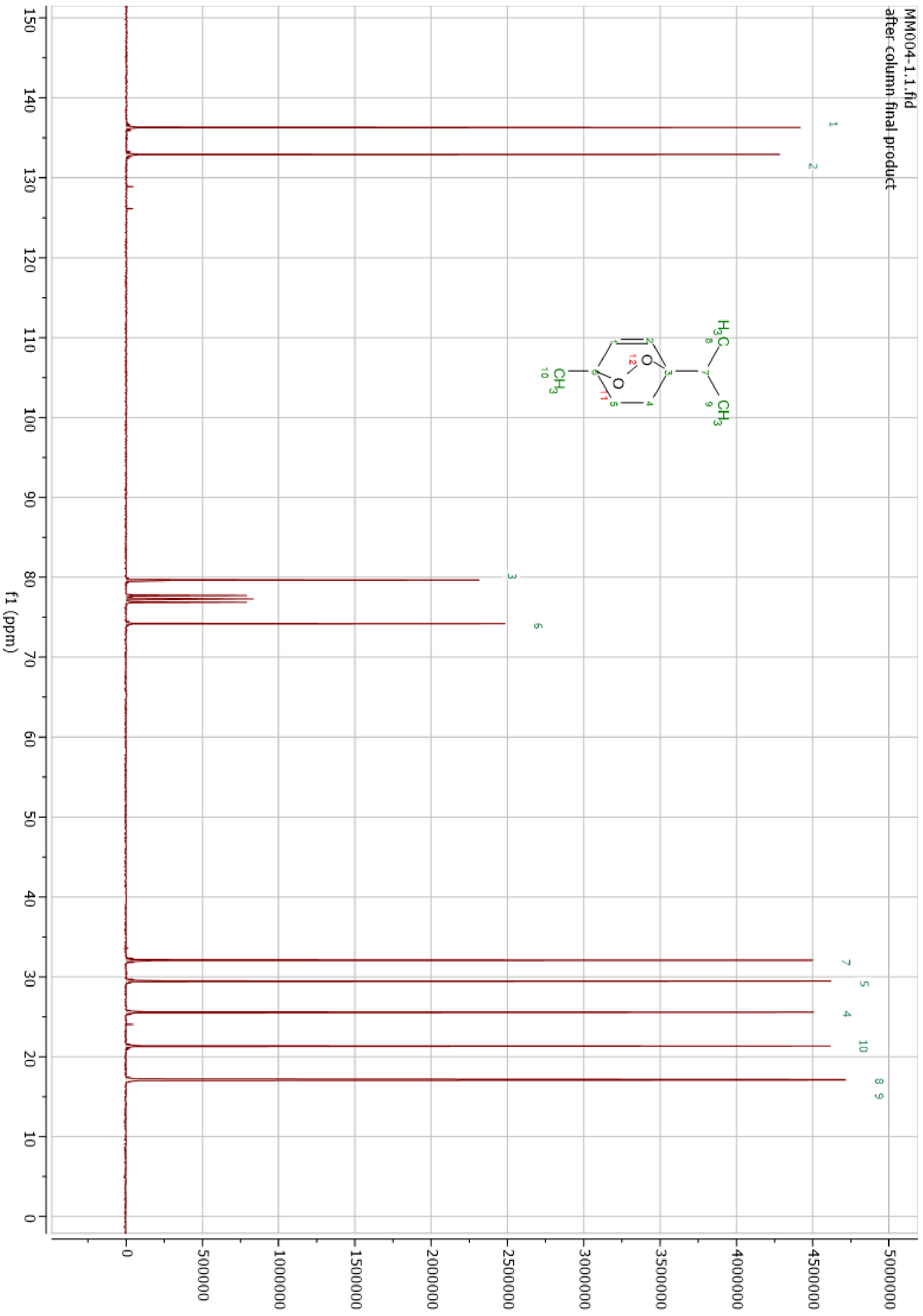

Figure S10: ^{13}C-NMR spectrum of alpha-terpinene

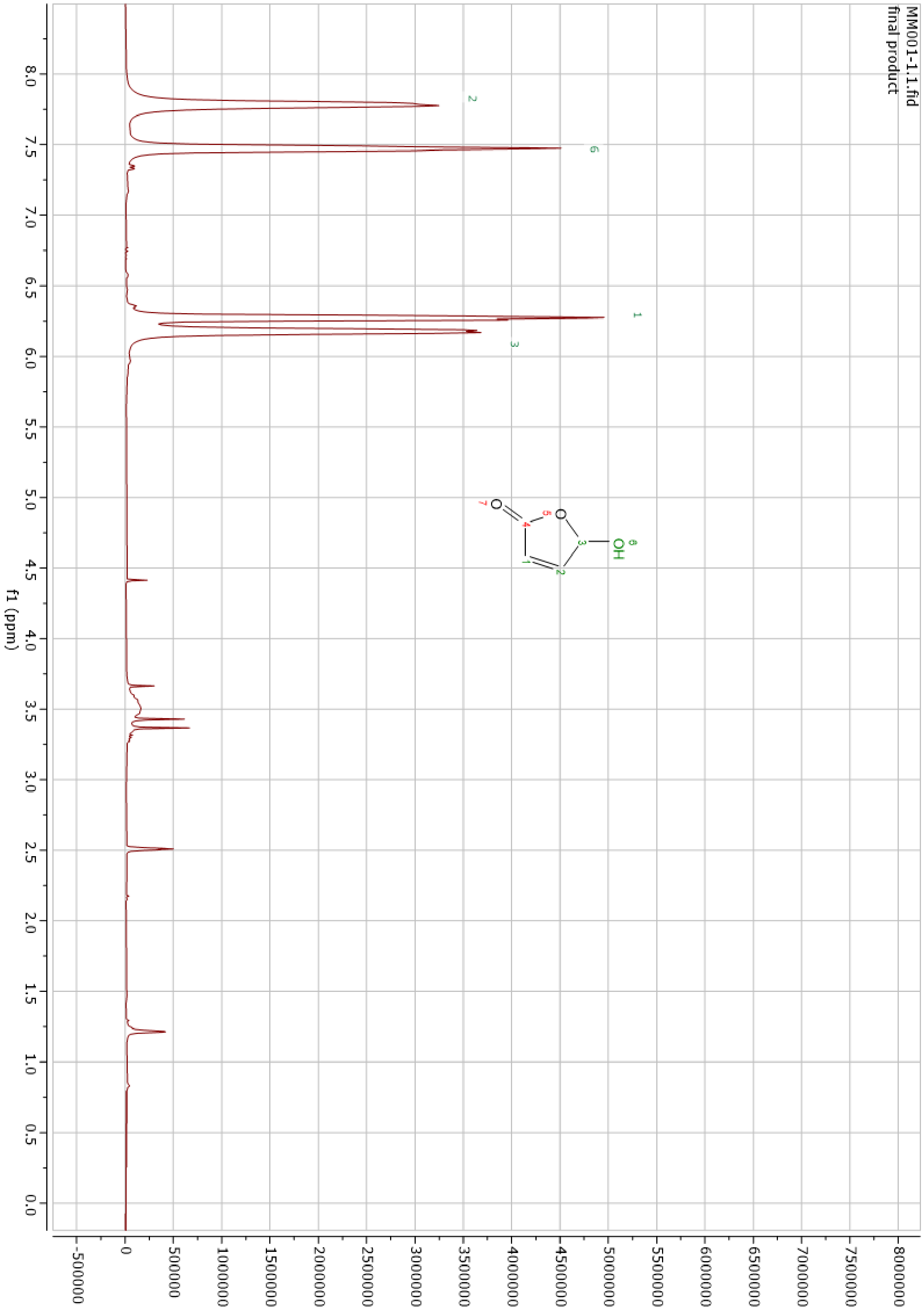

Figure S11: ^{1}H-NMR spectrum of 5-hydroxyfuran-2(5H)-one

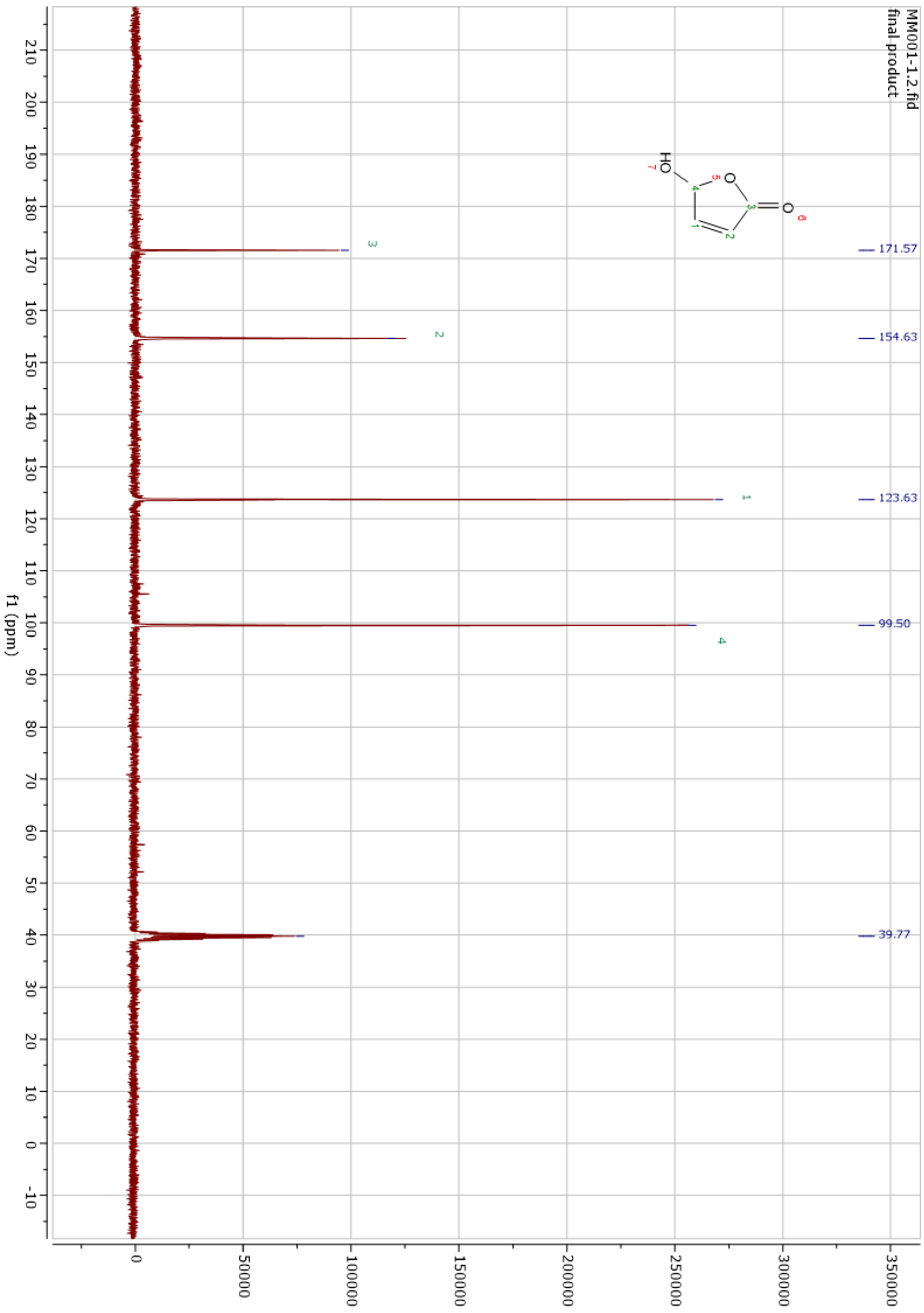

Figure S12: ^{13}C-NMR spectrum of 5-hydroxyfuran-2(5H)-one

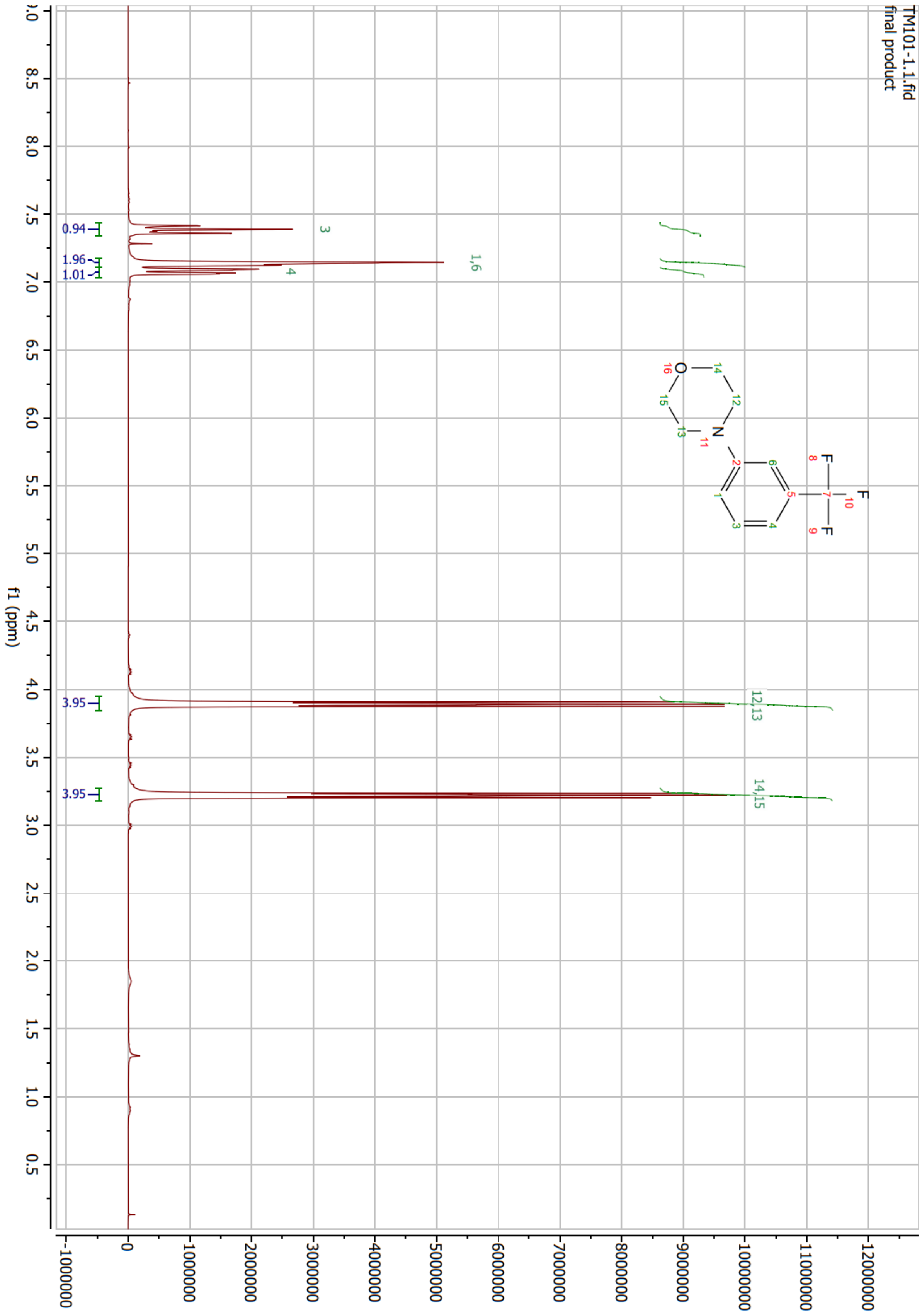

Figure S13: ^{1}H-NMR spectrum of 4-(3-(trifluoromethyl)phenyl)morpholine

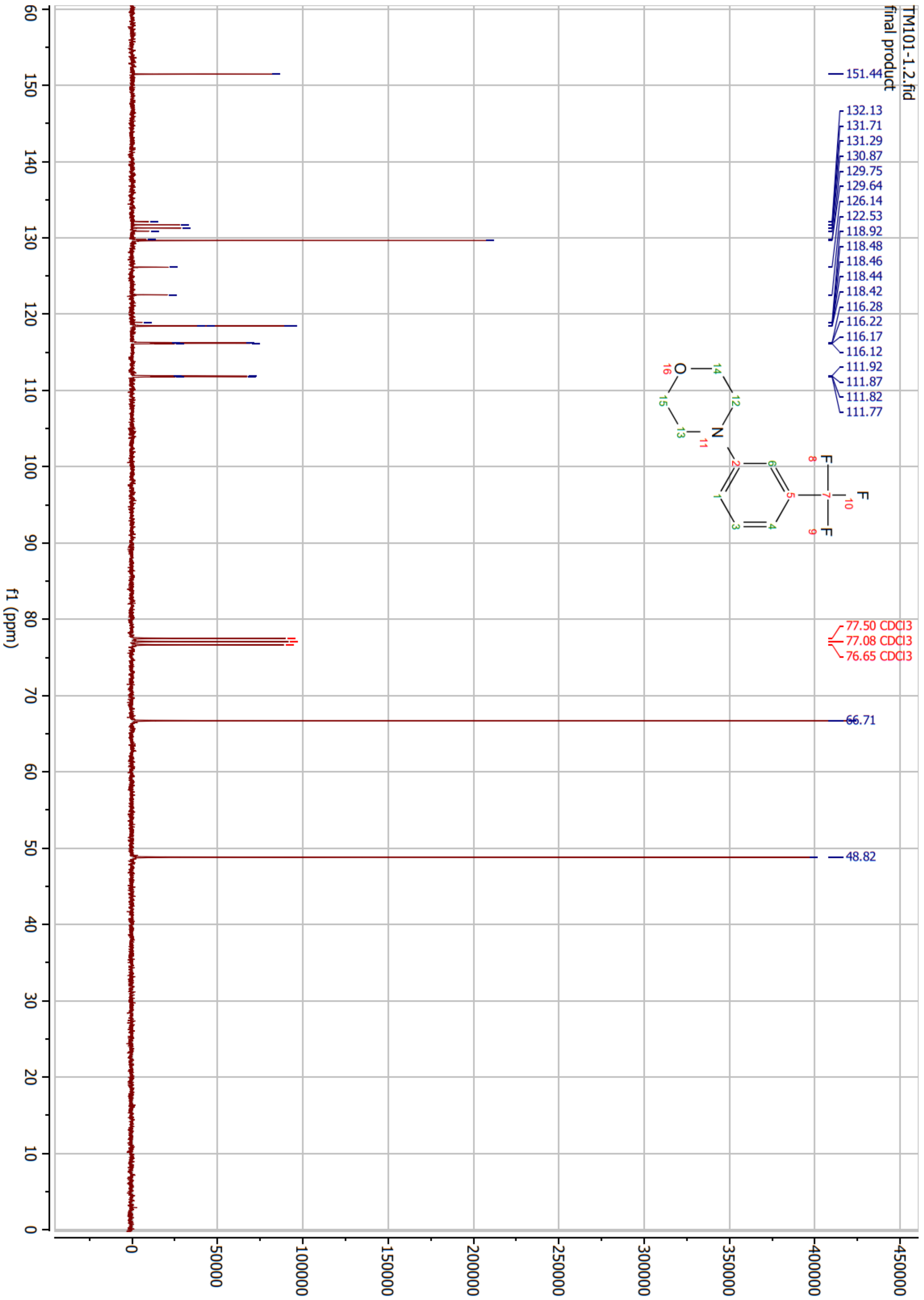

Figure S14: ^{13}C-NMR spectrum of 4-(3-(trifluoromethyl)phenyl)morpholine

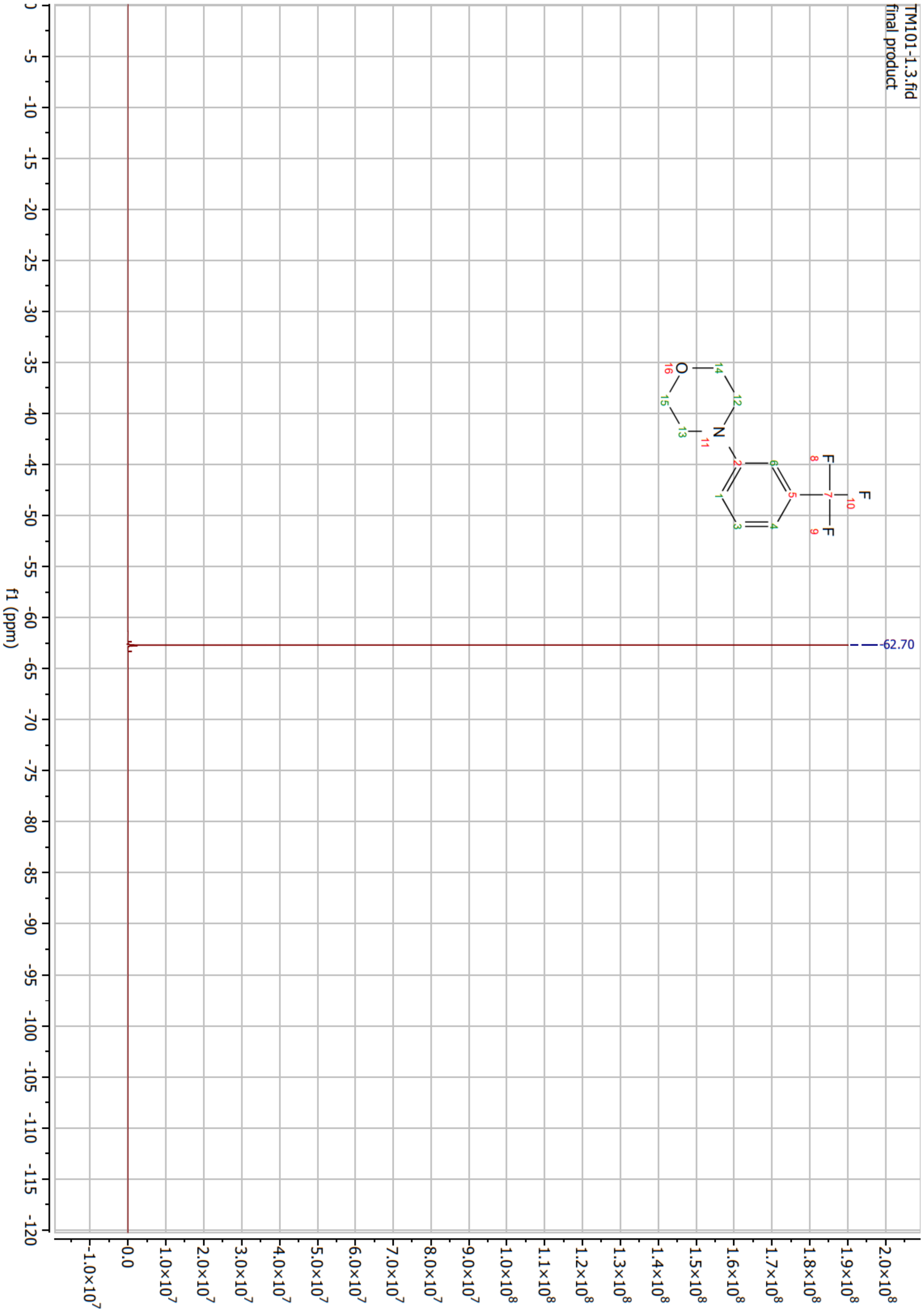

Figure S15: ^{19}F-NMR spectrum of 4-(3-(trifluoromethyl)phenyl)morpholine

References

(1) Kuijpers, K. Scaling and Automation of Photochemistry in Continuous Flow, 2020.

(2) Masson, T. M.; Zondag, S. D. A.; Kuijpers, K. P. L.; Cambié, D.; Debije, M. G.; Noël, T. Development of an Off-grid Solar-powered Autonomous Chemical Mini-plant for Producing Fine Chemicals. *ChemSusChem* **2021**. https://doi.org/10.1002/cssc.202102011.

(3) Verbunt, P. P. C.; Kaiser, A.; Hermans, K.; Bastiaansen, C. W. M.; Broer, D. J.; Debije, M. G. Controlling Light Emission in Luminescent Solar Concentrators Through Use of Dye Molecules Aligned in a Planar Manner by Liquid Crystals. *Adv. Funct. Mater.* **2009**, *19* (17), 2714–2719. https://doi.org/10.1002/adfm.200900542.

(4) Bruijnaers, B. J.; Schenning, A. P. H. J.; Debije, M. G. Capture and Concentration of Light to a Spot in Plastic Lightguides by Circular Luminophore Arrangements. *Adv. Opt. Mater.* **2015**, *3* (2), 257–262. https://doi.org/10.1002/adom.201400414.
